# Combined Metabolic and Functional Tumor Volumes on [^18^F]FDG-PET/MRI in Neuroblastoma Using Voxel-Wise Analysis

**DOI:** 10.3390/jcm12185976

**Published:** 2023-09-15

**Authors:** Maryanna Chaika, Simon Männlin, Sebastian Gassenmaier, Ilias Tsiflikas, Helmut Dittmann, Tim Flaadt, Steven Warmann, Brigitte Gückel, Jürgen Frank Schäfer

**Affiliations:** 1 Department of Diagnostic and Interventional Radiology, University Hospital Tuebingen, 72076 Tuebingen, Germany; 2 Department of Nuclear Medicine and Clinical Molecular Imaging, University Hospital Tuebingen, 72076 Tuebingen, Germany; 3 Department of Hematology and Oncology, University Children’s Hospital Tuebingen, 72076 Tuebingen, Germany; 4 Department of Pediatric Surgery and Pediatric Urology, University Children’s Hospital Tuebingen, 72076 Tuebingen, Germany

**Keywords:** high-risk neuroblastoma, PET/MRI, voxel-wise analysis

## Abstract

Purpose: The purpose of our study was to evaluate the association between the [^18^F]FDG standard uptake value (SUV) and the apparent diffusion coefficient (ADC) in neuroblastoma (NB) by voxel-wise analysis. Methods: From our prospective observational PET/MRI study, a subcohort of patients diagnosed with NB with both baseline imaging and post-chemotherapy imaging was further investigated. After registration and tumor segmentation, metabolic and functional tumor volumes were calculated from the ADC and SUV values using dedicated software allowing for voxel-wise analysis. Under the mean of thresholds, each voxel was assigned to one of three virtual tissue groups: highly vital (v) (low ADC and high SUV), possibly low vital (lv) (high ADC and low SUV), and equivocal (e) with high ADC and high SUV or low ADC and low SUV. Moreover, three clusters were generated from the total tumor volumes using the method of multiple Gaussian distributions. The Pearson’s correlation coefficient between the ADC and the SUV was calculated for each group. Results: Out of 43 PET/MRIs in 21 patients with NB, 16 MRIs in 8 patients met the inclusion criteria (PET/MRIs before and after chemotherapy). The proportion of tumor volumes were 26%, 36%, and 38% (v, lv, e) at baseline, 0.03%, 66%, and 34% after treatment in patients with response, and 42%, 25%, and 33% with progressive disease, respectively. In all clusters, the ADC and the SUV correlated negatively. In the cluster that corresponded to highly vital tissue, the ADC and the SUV showed a moderate negative correlation before treatment (R = −0.18; *p* < 0.0001) and the strongest negative correlation after treatment (R = −0.45; *p* < 0.0001). Interestingly, only patients with progression (*n* = 2) under therapy had a relevant part in this cluster post-treatment. Conclusion: Our results indicate that voxel-wise analysis of the ADC and the SUV is feasible and can quantify the different quality of tissue in neuroblastic tumors. Monitoring ADCs as well as SUV levels can quantify tumor dynamics during therapy.

## 1. Introduction

In neuroblastoma (NB), treatment and prognosis depend on the patient’s risk profile by age, evidence of amplification of the oncogene MYCN, distant metastases, and tumor grading [1,2]. Standard imaging used for risk stratification includes ultrasound (US), computed tomography (CT), magnetic resonance imaging (MRI), and 123I-metaiodobenzylguanidine (123I-mIBG) scan [3]. Image-defined risk factors before and during therapy using cross-sectional imaging allow the staging to be determined independently of the resectability [4]. Metabolic imaging such as PET/CT examinations with [^18^F]fluorodeoxyglucose (FDG) are indicated in MIBG-negative tumors and can also be used to assess the treatment response and the patient prognosis [5,6,7].

The diffusion-weighted imaging (DWI) by MRI measures the extent of water diffusion in vivo and is quantified by the apparent diffusion coefficient (ADC). The method is widely used in cancer imaging, providing valuable information for tumor detection, differentiation, and response to treatment. In other embryonal tumors like nephroblastoma, a good correlation between the ADC and grading, as well as the response to chemotherapy, has been shown in recent studies [8,9,10].

For several tumor entities, an inverse correlation between the ADC and FDG uptake as the SUV (standard uptake value) using mean or maximum/minimal values has been found, making superfluous information available from a clinical point of view [2,11,12,13,14]. To our knowledge, no published data for neuroblastoma exist in this context. However, global or single values cannot assess the tumor heterogeneity present in most cancers. Moreover, undifferentiated, poorly differentiated, and already mature tumor parts can occur side by side in NB [15]. Therefore, a precise tumor tissue assessment is particularly important for local therapy, as complete tumor resection is not possible in every case. Only a few studies have performed a voxel-wise evaluation of the whole tumor volume since an exact anatomical allocation of the two methods is necessary using simultaneous acquisition by PET/MRI [11,16]. In the authors’ opinion, PET/MRI is an important state of the art imaging modality for pediatric tumors, as tumor morphology and metabolic activity can be imaged in a “one-stop shop” examination with low radiation exposure.

This observational PET/MRI study aimed to evaluate the feasibility of a voxel-wise assessment of the ADC and the [^18^F]FDG SUV in neuroblastic tumors. For this purpose, metabolic and functional tumor volumes were quantified using software designed for detailed voxel-level analysis. Based on these data, each voxel was categorized into one of three virtual tissue groups. In addition, a multiple Gaussian distribution technique was used to generate three distinct clusters from the entire tumor volume. Furthermore, in this study we wanted to investigate whether the expected inverse correlation applies to all tumor fractions and what changes can result from chemotherapy.

## 2. Methods

### 2.1. Patients

The patients are a subcohort of our prospective observational PET/MR study (250/2014BO1). The inclusion criteria for the current analysis were histologically confirmed neuroblastoma, PET/MRI with [^18^F]FDG before and after induction chemotherapy or relapse chemotherapy, respectively, and reasonable image quality of the primary tumor region without severe artifacts. No additional sequences beyond standard of care were obtained. The corresponding institutional review boards approved the study. All patients and legal guardians provided written informed consent.

### 2.2. Image Acquisition

Examinations were conducted using a dedicated PET/MRI scanner (Biograph mMR^®^, Siemens Healthcare GmbH, Erlangen, Germany). To ensure accurate results, participants were required to have a normal fasting blood glucose level before receiving an intravenous injection of 2 mBq/kg [^18^F]FDG [17]. PET and MRI were started simultaneously approximately 60 min after tracer injection. PET events were detected for 4 min per bed position. PET data were reconstructed with a vendor-provided 3D-OSEM algorithm (iterations, 3; subsets, 21; matrix size, 256 × 256; voxel size, 2.8 × 2.8 × 2 mm^3^; Gaussian filter, 3 mm) [18]. PET/MRI were used for up to 5 bed positions.

After PET acquisition, the following sequences were used: 3-D T1-weighted spoiled gradient-echo sequence with Dixon-based fat-water separation, STIR coronal and axial, transversal T2-weighted turbo spin echo (TSE) and diffusion-weighted imaging DWI and T1 volumetric interpolated breath-hold examination (VIBE).

All sequence parameters are displayed in Table 1.

### 2.3. Data Processing

**Figure 1:**
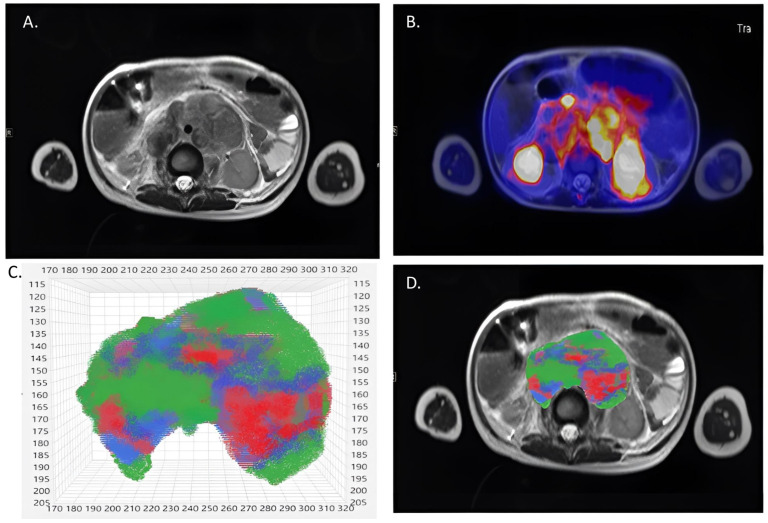
Example of tumor segmentation and clustering in a 3-year-old patient (Nr. 1) with retroperitoneal manifestation of neuroblastoma. (**A**) Axial T2-weighted image. (**B**) Axial fused image of the corrected PET dataset and the T2-weighted image. (**C**) After clustering, the voxels were assigned to one of three clusters: cluster 1  =  red, cluster 2  =  blue, cluster 3  =  green. (**D**) The volume of interest included total tumor volume.

The images were analyzed voxel-wise in the dedicated software Multiparametric Analysis (Siemens Healthcare). Using a non-rigid fusion function, the software processed and combined PET and T2-weighted images and ADC maps acquired on the hybrid PET/MR scanner. Since the resolution of PET images is lower than that of ADC maps and T2-weighted images, all MR images were calibrated to 0.7 × 0.7 × 5 mm^3^. Anatomical correlations were confirmed independently by two readers: one with 2 years of experience (M.C.) and the other a board-certified pediatric radiologist with 30 years of experience and a sub-specialization in pediatric radiology (J.F.S.). The volumes of interest (VOIs) of the primary tumor mass were outlined on the T2-weighted images, enclosing both necrotic and solid parts. Major blood vessels and hematomas were excluded. A tumor segmentation example is displayed in Figure 1.

### 2.4. Data Analysis

The voxel volume was 2.45 mm^3^ or 0.00245 mL. For each voxel, the software prototype (Multiparametric Analysis Siemens Healthcare) generated values of ADC and SUV. These data were transferred into statistical software (JMP^®^ software version 16.1 from SAS Institute Inc., Cary, NC, USA) for further processing and evaluation. The tumor volumes before and after therapy of each patient were calculated by multiplying the number of voxels by the voxel volumes. The mean, median, and 95th/5th percentiles of the ADC and the SUV of each tumor at both time points were computed from these raw data.

Thresholds were set based on previous studies that defined the cutoff between malignant and low vital tissues to 2.5 for SUV and 1250 × 10^−6^ mm^2^/s for ADC [19,20]. Voxels with ADC values ≤ 50 × 10^−6^ mm^2^/s were defined as outliers and excluded from the analysis. Accordingly, four groups of voxels were identified: (1) voxels with high SUV and low ADC, (2) with high SUV and high ADC, (3) with low SUV and low ADC, and (4) with low SUV and high ADC. Using these thresholds, each voxel was assigned to one of three virtual tissue groups: highly vital (v) (SUV > 2.5 and ADC < 1250 × 10^−6^ mm^2^/s), low vital (lv) (with SUV < 2.5 or ADC > 1250 × 10^−6^ mm^2^/s), and equivocal (e) with ADC ≥ 1250 × 10^−6^ mm^2^/s and SUV ≥ 2.5 or ADC ≤ 1250 × 10^−6^ mm^2^/s and SUV ≤ 2.5 mm^2^/s.

**Figure 2:**
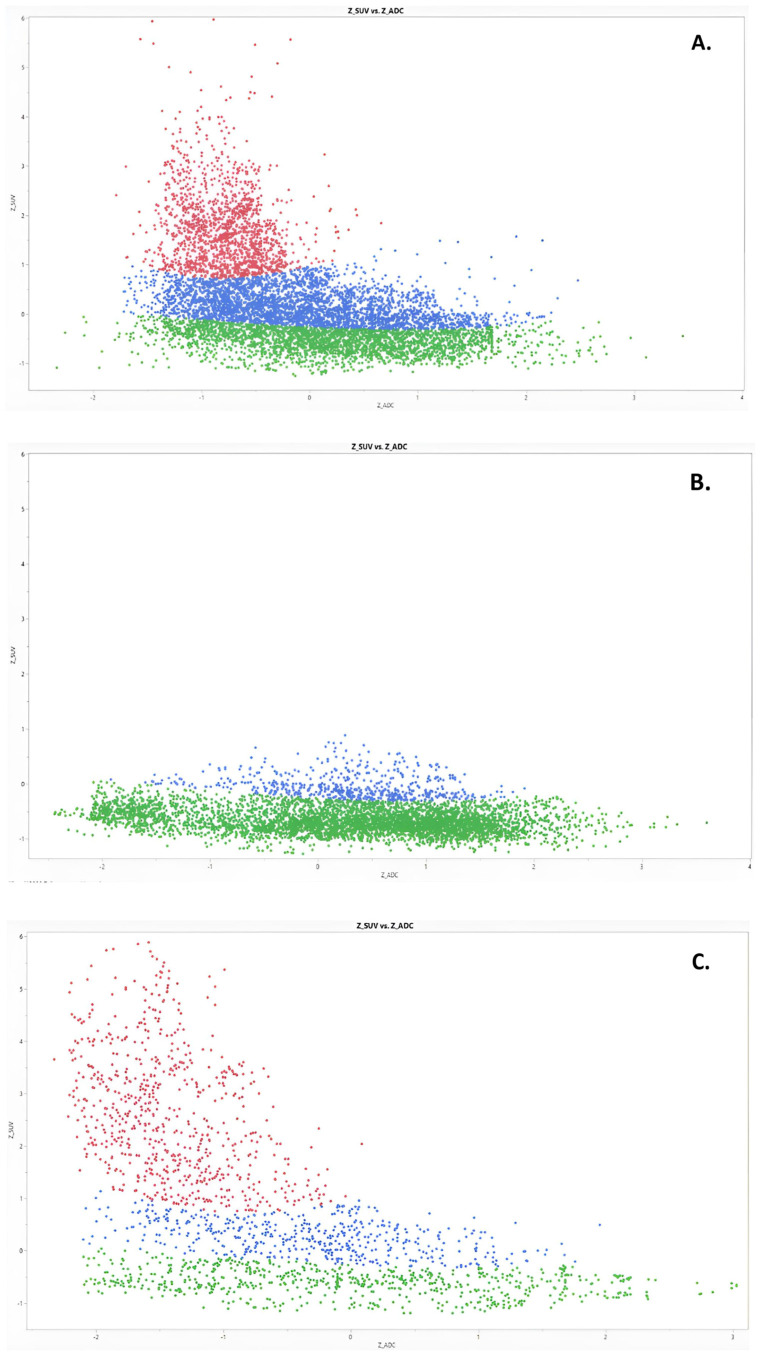
Proportion and distribution of the three clusters in total and separately for each patient before and after chemotherapy: (**A**) in total before therapy, (**B**) therapy response, (**C**) progressive disease. Red—“vital” cluster, blue—“equivocal”, green—“low vital”.

Based on the four groups described above, 3 clusters (“vital”, “equivocal”, and “low vital”) were generated in the statistical program used: for all patients before chemotherapy, for patients with a response to chemotherapy, and for patients with progression under chemotherapy. ADC- and SUV-dependent voxel distribution in these clusters are shown in Figure 2.

To avoid aberrance effects on results due to variable tumor sizes interindividual and by the same tumor before and after chemotherapy, each dataset was reduced or extrapolated to the same volume of 1000 voxels. Obtained ADC and SUV values were normalized collectively using the following formula:

$$x_{norm}=\frac{x-mean\;\left(x\right)\;}{std\;dev\;\left(x\right)}$$

As reported in previous studies [21,22], a voxel-based approach for [^18^F]FDG-PET and DWI was applied using the Gaussian mixture model (GMM). The collective dataset was subjected to fitting three Gaussian distributions, and the points of intersection between the subsequent two Gaussian distributions were designated as the demarcation thresholds for segmenting voxel-level data into distinct tumor subregions.

### 2.5. Statistical Evaluation

The Wilcoxon signed-rank test for paired data was used to compare mean SUV and ADC values before and after chemotherapy. Changes in the sub-volume (v, lv, e) after therapy were evaluated using Pearson’s correlation coefficient (point-biserial correlation) and linear regression. A threshold of *p* < 0.05 was defined to be statistically significant. JMP^®^ 16.1 (SAS Institute Inc., Cary, NC, USA) was used for all statistical evaluations.

## 3. Results

Out of 43 PET-MR examination results with an NB diagnosis, 16 diagnostic datasets from eight patients (each before and after chemotherapy), including three females, a mean age of 4 ± 2 years, and ages ranging from 1 to 10 years, were included according to the inclusion criteria. The examinations without follow-up and detectable lesions or with poor image quality (artifacts) were excluded. Three patients had NMYNC-amplified tumors, two tumors were MiBG-positive, and three were ALK-amplification positive. Response to therapy was demonstrated in six patients and a progressive disease was demonstrated in two. The patient’s characteristics are shown in Table 2 and Table 3.

**Figure 3:**
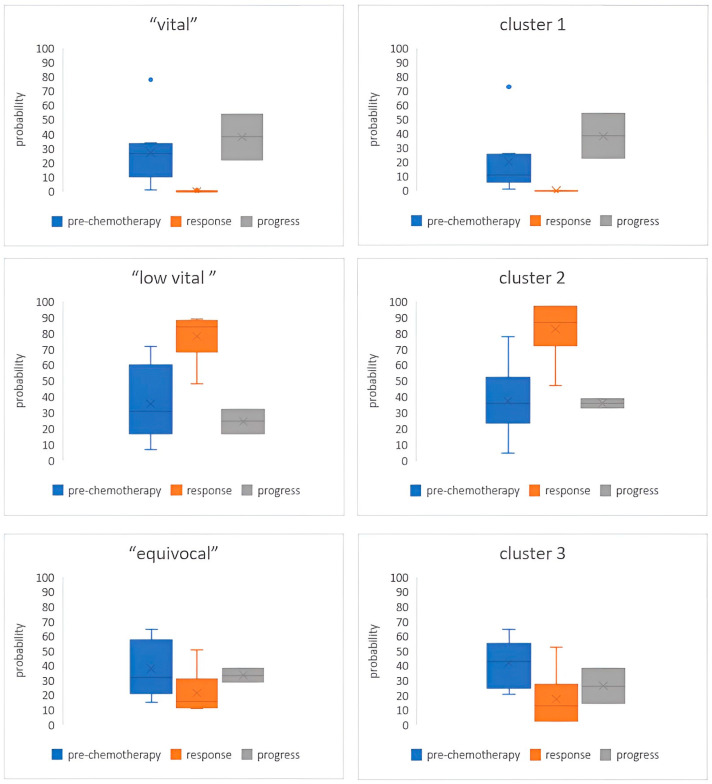
Proportion of voxels in three tissue groups (left) and clusters (right). Cluster 1 “malignant”, cluster 2 “low vital”, cluster 3 “equivocal”. There is a similar distribution between the respective clusters and tissue groups before and after chemotherapy: there was a significantly increase in the proportion of the “vital tissue” group in patients with progressive disease and a decrease in the “low vital tissue” group and in the “equivocal tissue” group. For patients responding to chemotherapy, the “low vital tissue” increased and the “vital tissue” decreased significantly. Interestingly, the “equivocal tissue” group also decreased significantly (*p* < 0.05).

The total voxel number of 655,204 pre-chemotherapy changed to 43,738 from response assessment post-chemotherapy and 163,559 in the case of progressive disease. The proportion of total tumor volumes, which was calculated from the voxel number and volume of the single voxel, was 1605 mL at baseline, 318 mL after treatment, and 190 mL as the disease progressed, respectively. The proportion of tumor volumes were 26.3%, 35.8%, and 37.9% (vital, low vital, equivocal) at baseline, 0.03%, 65.7%, and 34.3% (v, lv, e) after treatment at the response assessment, and fell to 41.8%, 24.9%, and 33.3% (v, lv, e) when the disease progressed during chemotherapy, respectively. In patients with progressive disease, there was a significant increase in the proportion of the “vital tissue” group from an average of 26.3% to 41.8% (*p* < 0.05), a decrease in the “low vital tissue” group from 35.8% to 24.9% *(p* < 0.05), and a decrease in the “equivocal tissue” group from an average of 37.9% to 33.3% (*p* < 0.05). For patients responding to chemotherapy, the “low vital tissue” increased from 35.8% to 65.7% (*p* < 0.05) and the “vital tissue” decreased from 26.3% to 0.03% (*p* < 0.05). Interestingly, the “equivocal tissue” group also decreased from 37.9% to 34.3% (*p* < 0.05). These results are depicted in Table 4 and Figure 3.

The qualitative cluster analysis used three Gaussian mixtures in Gaussian mixture model clustering for all datasets. The clusters’ distributions at baseline, by the response assessment and by progressive disease, are shown in Figure 3. When response was assessed after chemotherapy, “malignant” cluster 1 (red) decreased from a mean of 42.3% to 0.03% (*p* < 0.001). Interestingly, there was no significant decrease in this cluster by progressive disease (*p* = 0.31). In contrast, the “low vital” cluster 2 (green) response score increased from a mean of 25.3% to 69.6% (*p* < 0.05), but there were also no significant changes in this cluster as the disease progressed (*p* = 0.42). Figure 3 shows a comparison of the three clusters and the virtual tumor volume in all patient groups.

Mean overall SUV was significantly reduced in responders after chemotherapy, from 1.75 (SD 0.92) to 0.97 (SD 0.28) (*p* < 0.001), and also increased significantly to 2.26 (SD 1.52) in progressive disease (*p* < 0.001). Mean ADC also changed significantly from 1159 (SD 417) [10^−6^ mm^2^/s] at baseline to 1402 (SD 511) [10^−6^ mm^2^/s] at response and to 928 (SD 299) [10^−6^ mm^2^/s] at disease progression.

In voxel-wise analysis, the ADC and SUV values showed a negative correlation in all patients before chemotherapy and at disease progression (R = −0.32, −0.41; *p* < 0.0001) in the cluster, which contained more “malignant” voxels. In contrast, the ADC and SUV values correlated weakly positively in all patient groups in the cluster that contained low vital voxels (R = 0.326, 0.295; *p* < 0.0001). No correlation was found in clusters with equivocal voxels (R = 0.005, *p* > 0.05).

## 4. Discussion

The results of our study indicate that voxel-wise analysis of the ADC and the SUV is possible with simultaneous acquisition of MR and PET data to quantify the different quality of tissue in neuroblastic tumors. Based on this measurement, it is also possible to conclude the dynamics of changes in these parts after therapy.

Hybrid PET/MR imaging can provide functional and anatomic information in combination with a reduction in radiation dose, investigation time, and improvement of outcome after surgical tumor resection. Additionally, several studies demonstrated that FDG-PET-MRI is a promising modality in pediatric imaging for separating tumor sub-volumes, such as vital tumor tissue from necrosis or less malignant tumor parts [23,24,25,26,27,28]. The study from Surov et al. indicated that the correlation of SUV and ADC values can be used to estimate the proliferation potential of uterine cervical cancer [26]. Moreover, PET-MRI has shown to be a prognostic tool for the histological grading and survival in patients with gliomas [28]. In our study, mean SUV was significantly reduced in patients with response to chemotherapy and it was significantly increased from the progression of disease. Previous studies showed that the extension of high metabolic components in neuroblastoma could predict the progression and determine tumor burden [29]. In a study by Männlin and colleagues was demonstrated that the differing distribution of sub-volumes before and after therapy may hold prognostic significance in rhabdomyosarcoma [23]. In the present study, the simultaneous acquisition of the SUV in [^18^F]FDG and the ADC in DWI for voxel-wise analysis of neuroblastoma tumor sub-volumes in PET/MRI was investigated for the first time.

The increase in ADC values was determined after chemotherapy in patients with response assessment. In these patients, the mean ADC was significantly higher (*n* = 6) compared to baseline (*n* = 8) and progressive disease (1402 ± 511 versus 1159 ± 417 and 928 ± 338 × 10^−6^ mm^2^/s; *p* < 0.05). These findings are similar to previous investigations in other tumor entities [8,23]. Peschmann et al.’s study has shown that high malignant neuroblastoma showed lower ADCs than non-malignant neuroblastic tumors [9]. In ADC histogram analysis of neuroblastomas, MYCN-amplified neuroblastomas had statistically significant higher maximum ADCs and lower minimum ADCs [24]. Therefore, monitoring ADC levels can quantify tumor dynamics during therapy [25].

Several studies have demonstrated an inverse correlation between the SUV and the ADC in malignant lesions of the cervix and bronchial carcinoma [12,13,30] and in non-osseous and osseous tumors using FDG-PET/MRI [14]. In head and neck cancer, it has been shown that the SUV and the ADC correlate with different histopathological findings and, therefore, can be used as complementary biological markers [31]. Schmidt et al. also showed a strong reverse correlation between the ADC and the SUV in pulmonary lesions [16].

Our results are comparable with the study from Sun et al., who had already shown that PET/MR datasets can be used to evaluate PET and DWI based on sub-volume analysis, regardless of tumor cell type [32]. The expected negative correlation of low ADC and high SUV in all tumor volumes represents superfluous information [14]. Only patients with progression (*n* = 2) under therapy had a relevant part of the “vital” cluster with the strongest negative correlation of low ADC and high SUV. These findings may be helpful in several clinical settings. First, the diagnostic accuracy of biopsies can be increased by targeting the device to compartments containing vital tumor tissue. Secondly, non-invasive assessment of tumor necrosis can be used to monitor the response to treatment [33]. And third, furthermore, this can also be used for more precise surgical planning and dose adjustment for targeted radiotherapy.

A significant change in the proportion of voxels before and after treatment was determined in this study. Under the mean of thresholds, the ratio of the so-called vital tumor tissue decreased at response and increased with progressive disease. Appropriately, the proportions of clusters in the Gaussian distribution model showed similar dynamics after treatment. Moreover, the number of “vital” voxels in “malignant” cluster 1 decreased by the response assessment and increased in patients with progressive disease. The proportions of “equivocal” voxels in cluster 3 did not change significantly after therapy, both in patients with response and with progress. Conversely, the “low vital” tissue group in cluster 2 increased in patients in response to the chemotherapy.

Therefore, using voxel-wise analysis in a Gaussian mixture distribution allows us to make conclusions about tumor parts with varying degrees of malignancy. These findings are significant in heterogeneous tumors, where only small samples of the lesion can be evaluated due to biopsy. We used the thresholds based on previous studies that defined the cutoff between malignant and low vital tissues to 2.5 for the SUV and 1250 for the ADC [20]. However, no data are available for thresholds separating malignant from benign parts in neuroblastoma.

Our study has some limiting factors: first of all, the low number of cases should be mentioned. However, comparable MR datasets before and after chemotherapy have also resulted in a reduced number of patients. The positive correlation in “equivocal” voxels could also be due to cell differentiation or inflammatory/necrotic processes. In our study, no histological correlates were available to correlate with our SUV and ADC scores. Nevertheless, PET/MR imaging might add complementary information of PET and DWI based on the sub-volume analysis, regardless of the tumor cell type.

In conclusion, this study has shown the feasibility of voxel-wise analysis of the ADC and the SUV in the simultaneous acquisition of MR and PET data, which has also been shown in previous studies of other tumor entities [34]. The voxel-wise correlation of the SUV and the ADC provides promising results in differentiating diverse tumor parts. However, further investigations in larger cohorts with various clinical settings are needed to validate this hypothesis.

## Figures and Tables

**Table 1:** Acquisition parameters in examination protocol.

	Dixon	STIRcor	T2-TSE	STIRax	DWI
TE (echo time) [ms]	1.23/2.46	78	100	81	60
TR (repetition time) [ms]	3.6	6400	3500	4500	6000
bandwidth [Hz/px]	965	383	260	220	1860
matrix size [px]	79 × 192	256 × 256	256 × 300	197 × 384	108 × 192
resolution [mm^3^]	4.1 × 2.6 × 2.6	1.5 × 1.5 × 4	1.25 × 1.25 × 5	1.2 × 0.83 × 5	2.6 × 2.6 × 5
excitation angle [°]	10	120	90	120	90
inversion time [ms]		200		220	
b-values [mm^2^/s]					50 and 800

**Table 2:** Patients′ characteristics.

Patients	8
Sex	5 male, 3 female
Age:	
Mean age ± SD	4 ± 2 years
Range	1–10 years
Histology:	
MYNC-positive	*n* = 4
MiBG-positive	*n* = 3
ALK-amplification-positive	*n* = 2
Risk group stratification:	
High risk	5
Intermediate risk	2
Low risk	1

**Table 3:** Each patient’s characteristics and cluster distribution at baseline and after treatment.

Patient	Sex	Age	Stage/Risk	Genetic, EFS/OAS	Baseline	Post-Chemo
1	male	3	IV./high	-	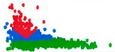	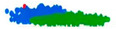
2	female	10	IV./high	ALK-positive	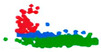	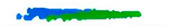
3	male	4	IV./high	ALK-positive	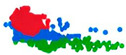	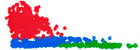
4	female	3	IV./high	N-MYC-positive	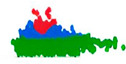	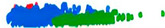
5	male	1	III./low	ALK-positive	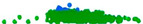	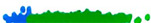
6	male	4	IV./high	N-MYC-positive	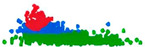	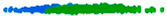
7	male	5	IV./high	-	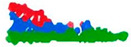	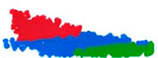
8	female	5	IV./high	N-MY-Cpositive	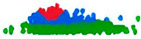	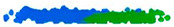

**Table 4:** Mean values of tumor volumes as well as of ADC and SUV values.

	Before Treatment	Response (*n* = 6)	Progress (*n* = 2)
ADC mean ± SD	1159 ± 417	1402 ± 511	928 ± 338
SUV mean ± SD	1.75 ± 0.92	0.97 ± 0.28	2.26 ± 1.52
Median Volume (total) [mL]	1605	318	190
vital	26.3%	0.03%	41.8%
low vital	35.8%	65.7%	24.9%
equivocal	37.9%	34.3%	33.3%

## Data Availability

Data available on request due to restrictions privacy and ethical. The data presented in this study are available on request from the corresponding author.
